# On the Action of a Secretion Obtained from the Medical Leech

**Published:** 1884-11

**Authors:** 


### On the Action of a Secretion Obtained From the Medicinal Leech.

Prof. John B. Haycraft has given in the Proceedings of the Royal Society some very interesting results of his investigations into this subject. It has long been a difficult problem to solve in the study of the coagulation of the blood, as to why the blood stripped from the leech after his application to man in "leeching" should remain fluid, and why there should be a tendency to the oozing of liquid blood from leech bites. Dr. Haycraft acted upon the idea that probably the leech secretes some ferment-containing juice, which antagonizes the blood ferment, thereby preventing coagulation within its body, and enough remaining around the edges of the wound to prevent the outflowing blood from clotting until the leech ferment is all washed away.

To decide this question, the gullets and buccal cavities of leeches were taken, cut in small pieces, and placed in a salt solution, resulting in an extract of leech of a faint greenish-yellow tint, and an alkaline reaction. This was found, when applied to freshly-drawn blood, to prevent coagulation. The extract was then boiled, but its characteristics remained unchanged, showing that it was not a ferment. Its active principle is insoluble in chloroform, ether, benzole, and alcohol, and could not be separated in a pure form.

The blood-coagulating ferment was then separated carefully from blood, and one part (*a*) treated with distilled water, the other part (*b*) with leech extract. On being added to hydrocele fluid, *a* produced a satisfactory clot, *b* not producing coagulation. Hence the action of the leech extract is to destroy the blood-coagulating ferment.

Fresh blood, mixed with leech extract, and placed under the microscope, showed that while coagulation was prevented, the corpuscles were not influenced, the red forming roleaux as usual, and the white exhibited the normal amœboid movements. Careful microscopical examination of the parts of the leech involved failed to show any glandular structure. The secretion is probably derived from the epithelial cells lining the sucker and buccal cavity; it may be that unicellular glands of the sucker share in its production.

The injection of this leech extract into the veins acts similarly to the injection of peptones in the non-coagulability of the blood, acting on dogs and rabbits alike. In the coagulation of milk it has no influence, but it hastens the coagulation of myosin, and probably when it causes loss of contractility in a muscle, it is due to this fact inducing rigor mortis, the essential phenomenon of which is clotting of the myosin.

